# Sedentary and physical activity time differs between self-reported ATLS-2 physical activity questionnaire and accelerometer measurements in adolescents and young adults in the United Arab Emirates

**DOI:** 10.1186/s12889-023-15881-8

**Published:** 2023-06-01

**Authors:** Ashokan Arumugam, Shima A. Mohammad Zadeh, Zina Anwar Zabin, Tamara Mohammad Emad Hawarneh, Hejab Iftikhar Ahmed, Fatema Shabbir Jauhari, Hanan Youssef Alkalih, Tamer Mohamed Shousha, Ibrahim M. Moustafa, Charlotte K. Häger

**Affiliations:** 1 Department of Physiotherapy, College of Health Sciences, University of Sharjah, P.O. Box: 27272, Sharjah, United Arab Emirates; 2 Neuromusculoskeletal Rehabilitation Research Group, RIMHS – Research Institute of Medical and Health Sciences, University of Sharjah, P.O. Box: 27272, Sharjah, United Arab Emirates; 3 Sustainable Engineering Asset Management Research Group, RISE - Research Institute of Sciences and Engineering, University of Sharjah, P.O. Box: 27272, Sharjah, United Arab Emirates; 4 Adjunct Faculty, Department of Physiotherapy, Manipal College of Health professions, Manipal Academy of Higher Education, Manipal, Karnataka India; 5 Department of Community Medicine and Rehabilitation – Physiotherapy Section, Umeå University, Umeå, SE-901 87 Sweden; 6 Faculty of Physical Therapy, Cairo University, Cairo, Egypt

**Keywords:** Accelerometry, Sensors, Wearable devices, ATLS-2, Validation, Agreement, Self-reported physical activity

## Abstract

**Background:**

Most young adults and adolescents in the United Arab Emirates (UAE) do not meet the established internationally recommended physical activity levels per day. The Arab Teen Lifestyle Study (ATLS) physical activity questionnaire has been recommended for measuring self-reported physical activity of Arab adolescents and young adults (aged 14 years to mid-twenties). The first version of the ATLS has been validated with accelerometers and pedometers (r ≤ 0.30). The revised version of the questionnaire (ATLS-2, 2021) needs further validation. The aim of this study was to validate the self-reported subjective sedentary and physical activity time of the ATLS-2 (revised version) physical activity questionnaire with that of Fibion accelerometer-measured data.

**Methods:**

In this cross-sectional study, 131 healthy adolescents and young adults (aged 20.47 ± 2.16 [mean ± SD] years (range 14–25 years), body mass index 23.09 ± 4.45 (kg/m^2^) completed the ATLS-2 and wore the Fibion accelerometer for a maximum of 7 days. Participants (n = 131; 81% non-UAE Arabs (n = 106), 13% Asians (n = 17) and 6% Emiratis (n = 8)) with valid ATLS-2 data without missing scores and Fibion data of minimum 10 h/day for at least 3 weekdays and 1 weekend day were analyzed. Concurrent validity between the two methods was assessed by the Spearman rho correlation and Bland-Altman plots.

**Results:**

The questionnaire underestimated sedentary and physical activity time compared to the accelerometer data. Only negligible to weak correlations (r ≤ 0.12; p > 0.05) were found for sitting, walking, cycling, moderate intensity activity, high intensity activity and total activity time. In addition, a proportional/systematic bias was evident in the plots for all but two (walking and moderate intensity activity time) of the outcome measures of interest.

**Conclusions:**

Overall, self-reported ATLS-2 sedentary and physical activity time had low correlation and agreement with objective Fibion accelerometer measurements in adolescents and young adults in the UAE. Therefore, sedentary and physical activity assessment for these groups should not be limited to self-reported measures.

## Background

Performing physical activities is vital in maintaining good health as it plays a large role in preventing cardiovascular diseases and reducing mortality rates [1–4]. For this reason, it is imperative that we maintain our physical health by participating in physical activities throughout the day and avoiding living sedentary lifestyles. Nowadays teens have been reported to often follow an inactive lifestyle [5]. Children and adolescents in the United Arab Emirates (UAE) do not reach the recommended amount of physical activity per day [6]. Physical activity report cards of children and youths in the UAE showed that only 25% of adolescents (aged 13–17 years) participated in physical education classes despite the governmental requirements for a minimum number of lessons per week. Moreover, only less than half of the adolescents met the recommended guidelines for screen time, and sedentary time further increased with age [7]. Directly monitoring the activity levels allows people to get an idea of how much or how little they are doing and may help adjusting their level of activity in order to improve or maintain their own health [8, 9]. Furthermore, measuring activity levels can also be used to survey the overall health of a population, which can be helpful in designing a health program for the benefit of the population or test the effectiveness of a health program [10, 11].

Generally, people struggle with incorporating physical activities in their daily routines as they have to adjust it according to their work and/or school schedules. In some cases, certain jobs allow people to be more active because of their work nature. Another aspect that can impact the activity level is the amount of leisure time given after work and school during the week [12–14]. The world-wide lockdown imposed following the COVID-19 pandemic had a great negative impact on many people’s daily physical activities, contributing markedly to the increasingly sedentary lifestyle adopted across the globe [15–19]. To counteract these negative effects, health policies need to be implemented. According to the revised policies in the UAE for the Sharjah Emirate, there are three official days off for the weekend effective from January 2022. This can result in two different scenarios: some people may like to spend their time participating in more physical activities such as playing sports or outdoor games while some people may like to spend time watching television or by participating in home-based activities. With the latter option, it is important to monitor activity during these days to ensure that activity levels do not drop significantly for teens and young adults with the introduction of the extra day of the weekend.

A fairly recent systematic review by Sharara et al. (2018) provides evidence for the lack of physical activity and also the prevalence of obesity in Arab countries [20]. In order to collect data for a large population, a self-reported questionnaire is the easiest way to collect, analyze and administer data. Each method has its advantages and disadvantages; therefore, the decision regarding which measure to use will be based on the purpose of the research, sample size, and availability of resources [21]. The Arab Teens Lifestyle Study (ATLS) questionnaire is commonly used to assess the physical activity levels, sedentary behavior, and dietary habits of teens and young adults in Arab countries since 2011. Al-Hazzaa et al. (2011) recommended the ATLS as a valid tool for estimating sedentary behavior and physical activities in individuals with a mean age of 16.1 ± 1.1 years [21]. Recently, this questionnaire has been revised and renamed as ATLS-2 [22]. The age group of the users of this questionnaire ranges from 14 to > 30 years [21–26]. A study by Al-Hazzaa et al. in 2011 [21] reported comparable estimates of self-reported physical activity levels with the ATLS and step counts measured by pedometers (r ≤ 0.30); however, the reported r value showed a weak correlation. Quantitative accelerometer-measured sedentary and physical activity data are further required to validate the self-reported measures of the ATLS-2 physical activity questionnaire. Previous studies have reported conflicting inconsistent findings between sedentary behavior and physical activity levels with self-reported questionnaires compared to accelerometer data [27–29].

Recent studies have used objective methods such as accelerometer devices to substantiate the validity and reliability of sedentary behavior and physical activity variables [30, 31] when measured for a period of 7 days [32]. The use of activity monitors can enhance participants attitudes toward cautiously monitoring their daily activities; therefore, these devices can be used to promote a healthy lifestyle for Arab teens [33]. Previously, the Actigraph accelerometer has been used for physical activity assessment of young adults in the UAE [34]; however, as per one study, its reliability in detecting moderate to vigorous intensity activities was not specifically evaluated [35]. The Fibion is a validated, new triaxial thigh-worn accelerometer that has been used in certain recent studies in the UAE [36, 37, 38]. There is no study yet reporting the validity of self-reported ATLS-2 physical activity questionnaire compared to a thigh-worn accelerometer measured sedentary and physical activity levels.

The aim of this study was to evaluate the concurrent validity of the self-reported sedentary and physical activity time of the ATLS-2 physical activity questionnaire compared to Fibion accelerometer-measured data in adolescents and young adults of the UAE. We hypothesized that self-reported sedentary and physical activity time of the ATLS-2 would show negligible/weak correlations with Fibion accelerometer-measured variables in adolescents and young adults.

## Methods

### Study design and setting

We used a cross-sectional design for this study on adolescents and young adults. Participants were recruited from the UAE’s public and private schools and universities between January and August 2022.

### Participants

One hundred and fifty physically active adolescents and young adults of both sexes, aged between 14 and 25 years, were enrolled from any of the UAE’s public and private schools and universities. According to a literature review of 114 studies reporting validity of self-reported questionnaires, 90% used a sample size ≥ 100. Moreover, the COSMIN guidelines recommend a sample size more than 100 for validation studies [39]. Therefore, 150 participants were deemed sufficient for this study [40]. Though the term “teen” has been used for referring to the instrument, the developers of the ATLS questionnaire have applied it to participants aged between 14 and mid-twenties [21–26]. Participants in the present study were excluded if they had any of the following pathological conditions: musculoskeletal, rheumatic, cardiovascular, or systemic conditions or any recent surgery affecting physical activity, sleep, and/or dietary patterns. Participants were recruited using advertisements placed on university/school notice boards, flyers, mobile applications (e.g., WhatsApp) and/or word of mouth. The Research Ethics Committee of the University of Sharjah reviewed the study proposal and approved it (REC-22-02-23-01-S). Participants and/or their parents read the information sheet and then informed consent was obtained from them (for adults) or their parents/guardians (for adolescents) before they were enrolled in the study.

### Research instrument

Anthropometric measurements were measured using a stadiometer for height assessment (Seca 213- Hamburg, Germany) and a body composition analyzer (Tanita HD 318 Tanita, Tokyo, Japan) for weight assessment. Sedentary behavior and physical activity were assessed using the ATLS-2 physical activity questionnaire (https://lh-hsrc.pnu.edu.sa/wp-content/uploads/2018/11/ATLS-Questionnaire-E-Revised-2018-1-1.pdf) and the Fibion device (Fibion Inc, Jyväskylä, Finland).

The ATLS-2 enables collecting and analyzing important lifestyle information from Arab teenagers and young adults [40]. The ATLS-2 questionnaire is used by adolescents and young adults to self-report variables including anthropometric measures, physical activity (with 34 items), sedentary behavior (with 4 items), sleep duration (with 2 items), and dietary habits (with 10 items). However, dietary questions were not included in the current study. The questionnaire is available in English and Arabic versions. Our participants were given the option to choose either version based on their choice.

### Procedure

The ATLS-2 questionnaire was filled out online by the participants via Google Forms before wearing the Fibion accelerometer. Sedentary and physical activity duration was assessed using the Fibion device affixed to the right anterior thigh. The participants were asked to wear the Fibion device for one week and remove it during any water-based activity since the device is not waterproof. The Fibion device was worn in the proximal third of the thigh as instructed by the official Fibion website. A non-allergic adhesive tape was used to secure the device to the body. The Fibion device can precisely measure and analyze different intensities of physical activities when it is placed on the anterior mid-thigh [37, 42].

### Accelerometer data processing

We used Fibion data processing techniques reported in earlier studies [20, 22] to analyze physical activity time. Fibion data, as well as each student’s age, sex, weight, and height, were submitted to the manufacturer’s website (www.fibion.com/upload). As a result, the web service provided explicit reports on the time, intensity, and energy expenditure of physical activity levels. Data from CSV files including minute-by-minute and day-by-day, data were retrieved and analyzed. A customized data fixer tool from the Fibion manufacturer was used to exclude standard night-time (11 pm to 7 am) from all participants to prevent conflation between night-time data with sedentary and upright time data [37].

For the data to be considered valid, a minimum of 10 h (600 min) per day for 3 weekdays and 1 weekend day must be collected [37]. Only sedentary behavior and physical activity time per day was included for analysis, after including the number of valid days of participation for each task.

Among the 150 participants who were initially enrolled in the study, 19 were excluded due to invalid data or technical difficulties with the devices during data collection, and 131 were thus included in the analysis. To mitigate the differences in wear time of the Fibion device between the participants, all the variables were normalized to 16 h of activities ([the time duration of each task*16]/the total wear time) [29, 43]. All the variables of interest (duration of walking, cycling, high intensity, moderate intensity and total activity), except for sitting/sedentary time (hours/16-hour day), extracted from the Fibion data were expressed as minutes/16-hour day. The ATLS-2 physical activity questionnaire data were obtained using Google sheets associated with the Google form customized for ATLS-2 data collection. Participants self-reported the duration of activities in minutes per r (24-hour) day. Some tasks mentioned in the ATLS-2 questionnaire such self-defense sport, household work, and dancing activities will fall into one of the three intensity types (light, moderate, and high). As the accelerometer devices cannot record the contextual factors of activity (e.g., self-defense, household, or dancing activity) and upper limb activities, the intensity of activity was considered while calculating the total activity time from the Fibion output. For ATLS-2 total activity time, a sum of walking, run/jogging, cycling, moderate intensity, high intensity, self-defense sport, household work and dancing activities was used.

### Statistical analysis

The Shapiro-Wilk tests were used to determine if the data were normally distributed. Concurrent validity between ATLS-2 and Fibion data was assessed using the Spearman’s Rho correlation coefficients as the data were not normally distributed. The criteria used for interpreting correlation coefficients are shown in Table 1 [36, 44]. The Bland-Altman plots and 95% limits of agreement were used to detect outliers and systematic/proportional bias. In the Bland-Altman plots, mean values were plotted against differences between Fibion and ATLS-2 measures (i.e., Fibion accelerometer-measured time – self-reported time with the ATLS-2 for each task). These plots included 95% limits of agreement (mean ± [1.96 * SD] where mean and SD are the mean and standard deviation of differences between Fibion and ATLS-2 measures, respectively). In addition, proportional bias in the data was assessed using linear regression analyses with difference and mean scores of the two methods (ATLS-2 and Fibion accelerometer data) and used as dependent and independent variables respectively [43]. A significance level < 0.05 was set for all analyses. Statistical analyses were performed with the IBM SPSS statistics version 28 (IBM Corp., Armonk, NY, USA).

**Table 1:** Criteria for interpreting the Spearman rho correlation coefficient

Correlation coefficient value range	Strength of correlation
0.00- 0.10	Negligible
0.10–0.39	Weak
0.40–0.69	Moderate
0.70–0.89	Strong
0.90- 1.00	Very strong

Note: These criteria were adapted from Schober et al.2018 [43].

## Results

Among 131 participants (age range: 14–25 years), 81% of the participants were non-Emirati Arabs (n = 106), 13% were Asians (n = 17), and 6% were Emiratis (n = 8). Other participants characteristics are summarized in Table 2.

**Table 2:** Study participants characteristics (n = 131)

Characteristics		Mean ± SD (except where indicated)	
Age (years)		20.47 ± 2.16	
Number (female, male)		131 (96, 35)*	
Height (cm)		165.53 ± 8.71	
Body mass (kg)		63.53 ± 14.31	
BMI (kg/$$ {m}^{2}$$)		23.09 ± 4.45	

BMI, body mass index; SD, standard deviation

*Number

All variables showed only negligible or weak and insignificant correlations between ATLS-2 and Fibion measurements. Overall, self-reported time with the ATLS-2 underestimated accelerometer-measured time for sedentary behavior and physical activities in the population studied. Study participants’ characteristics and the Spearman’s rho correlation coefficients correlating variables of the Fibion and ATLS-2 are presented in Tables 2 and 3, respectively. Most participants did not score any value for most activities (e.g., biking, dancing, and sports of various intensities) in the ATLS-2.

**Table 3:** Spearman rho correlation coefficients correlating variables of the Fibion and ATLS-2

Tasks (n = 131)	Duration, median (IQR)		Spearman rho	p values	Strength of correlation
	Fibion (per 16-hour day; night-time excluded)	Self-reported ATLS-2 (per day)			
Sitting/sedentary behavior time (hours)	11.04 (10.59–11.82)	5.00 (3.50–6.56)	-0.04	0.62	Negligible
Walking time (minutes)	88.11 (72.52-116.58)	30.00 (1.50–52.50)	0.05	0.55	Negligible
Cycling time (minutes)	0.01 (0.00-0.02)	0.00 (0.00–0.00)	-0.01	0.87	Negligible
High intensity activity (minutes)	1.76 (0.50–4.44)	0.00 (0.00-41.25)	-0.001	0.99	Negligible
Moderate intensity activity (minutes)	42.13 (31.16–66.06)	0.00 (0.00–30)	0.13	0.15	Weak
Total activity time (minutes)	276.32 (240.14-306.32)*	105.00 (69.50-166.25)**	0.03	0.76	Negligible

*Time spent: light intensity (< 3 METs [metabolic equivalents)]) + moderate intensity (3–6 METs) + high intensity activities (> 6 METs)

**Time spent: walking + walking/jogging + cycling + moderate intensity (e.g., volleyball, table tennis, bowling, badminton, aerobic dance or other similar activities) + high intensity (e.g., soccer, rugby, hockey, netball, basketball, handball, athletics, tennis, squash, etc.) + self-defense sport + house hold work + dancing activities

**Fig. 1:**
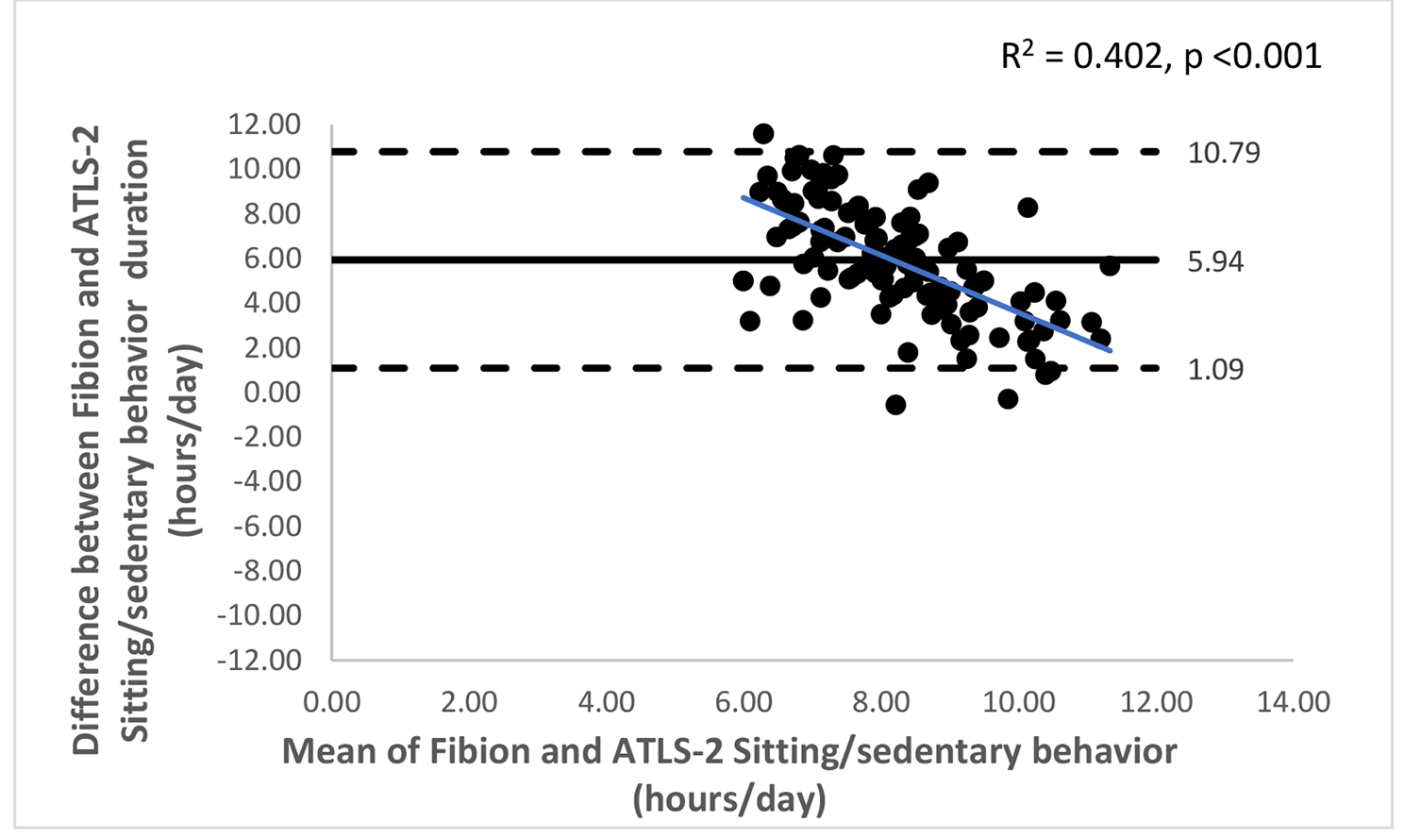
A Bland-Altman plot showing agreement between the Fibion accelerometer and ATLS-2 data for sitting time. The solid horizontal line represents bias (the mean of the differences between the two methods), while the horizontal dashed lines show upper and lower limits of agreement (bias ± (SD * 1.96)). The regression line is depicted in blue

**Fig. 2:**
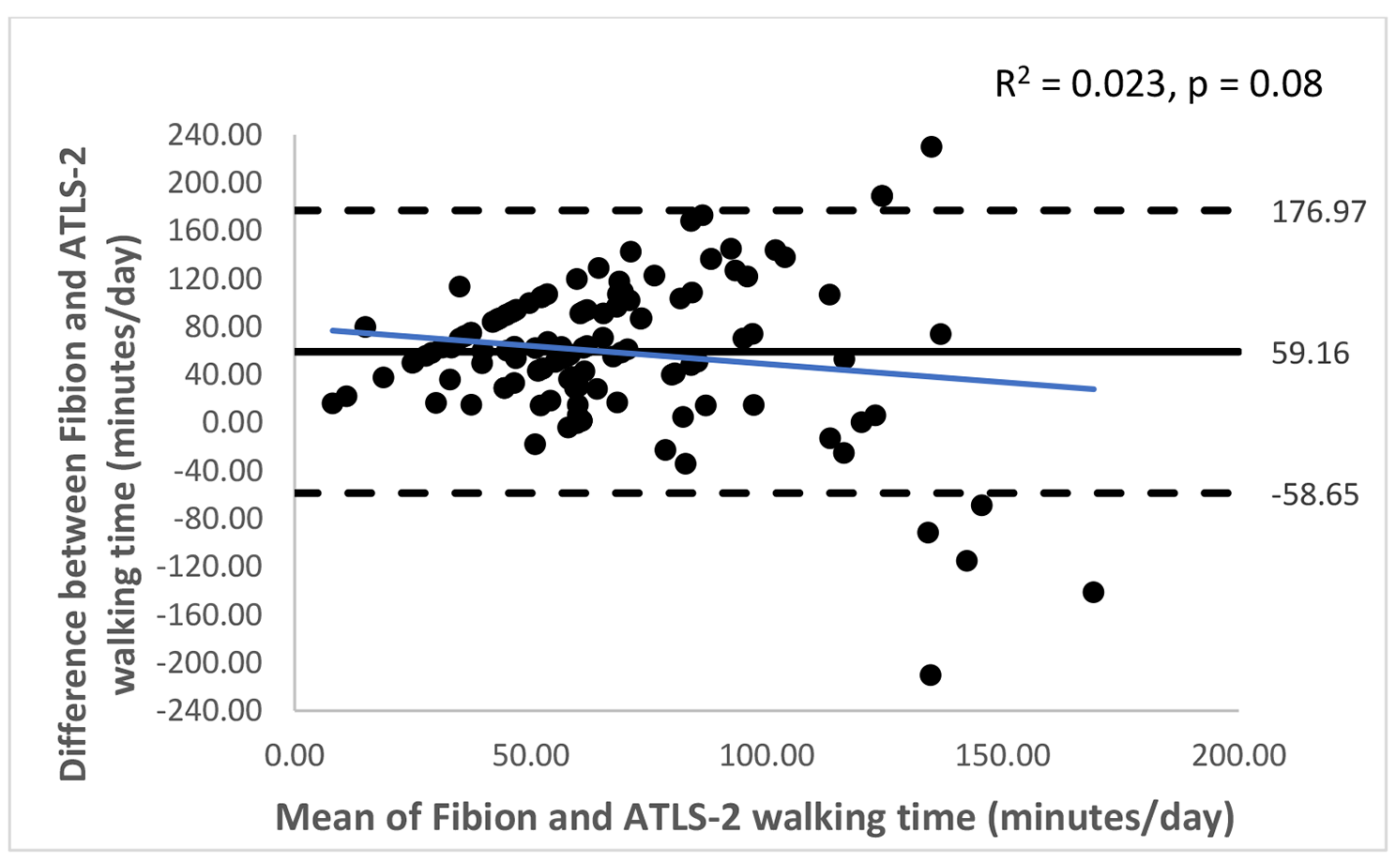
A Bland-Altman plot showing agreement between the Fibion accelerometer and ATLS-2 data for walking time. The solid horizontal line represents bias (the mean of the differences between the two methods), while the horizontal dashed lines show upper and lower limits of agreement (bias ± (SD * 1.96)). The regression line is depicted in blue

**Fig. 3:**
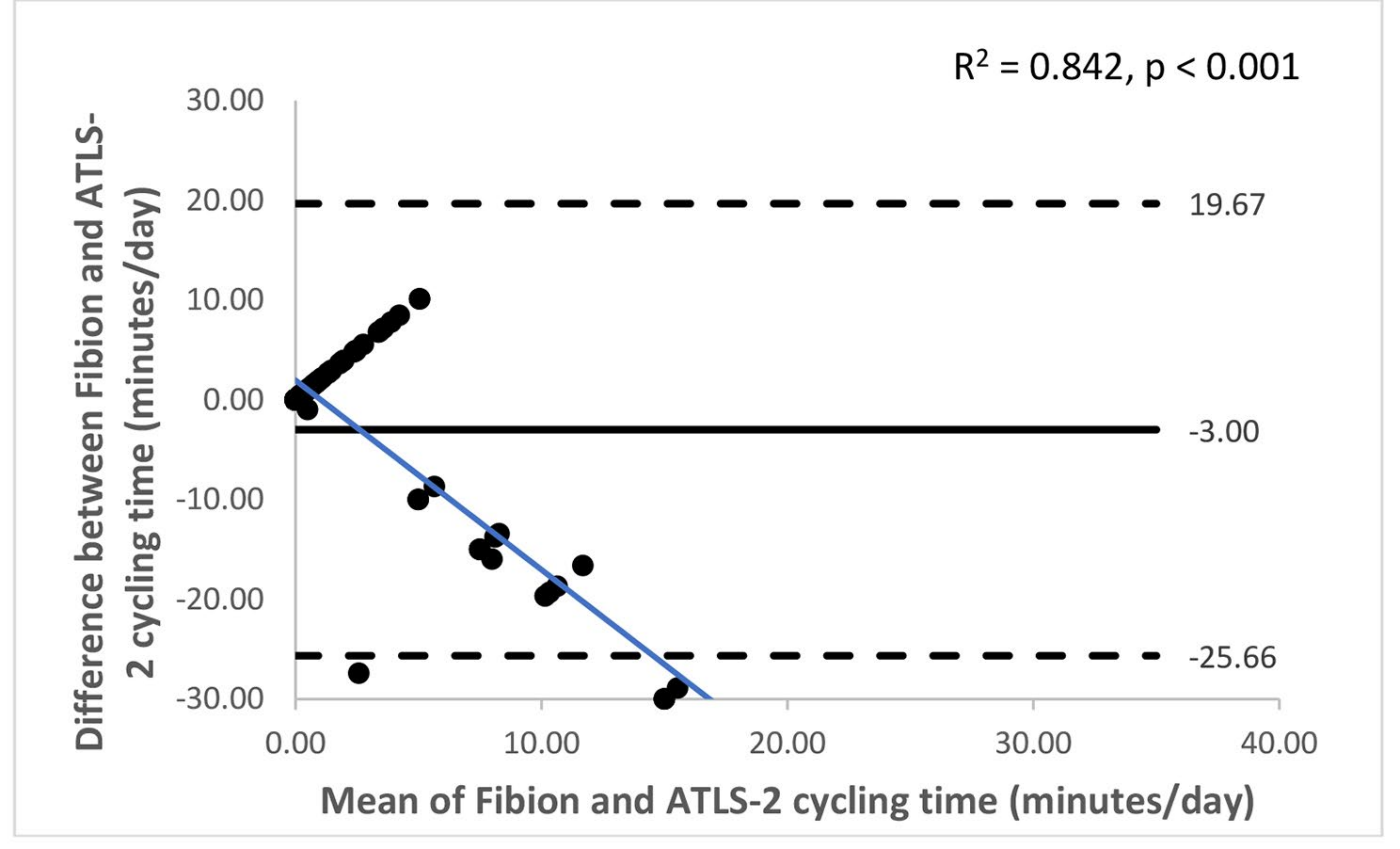
A Bland-Altman plot showing agreement between the Fibion accelerometer and ATLS-2 data for cycling time. The solid horizontal line represents bias (the mean of the differences between the two methods), while the horizontal dashed lines show upper and lower limits of agreement (bias ± (SD * 1.96)). The regression line is depicted in blue

**Fig. 4:**
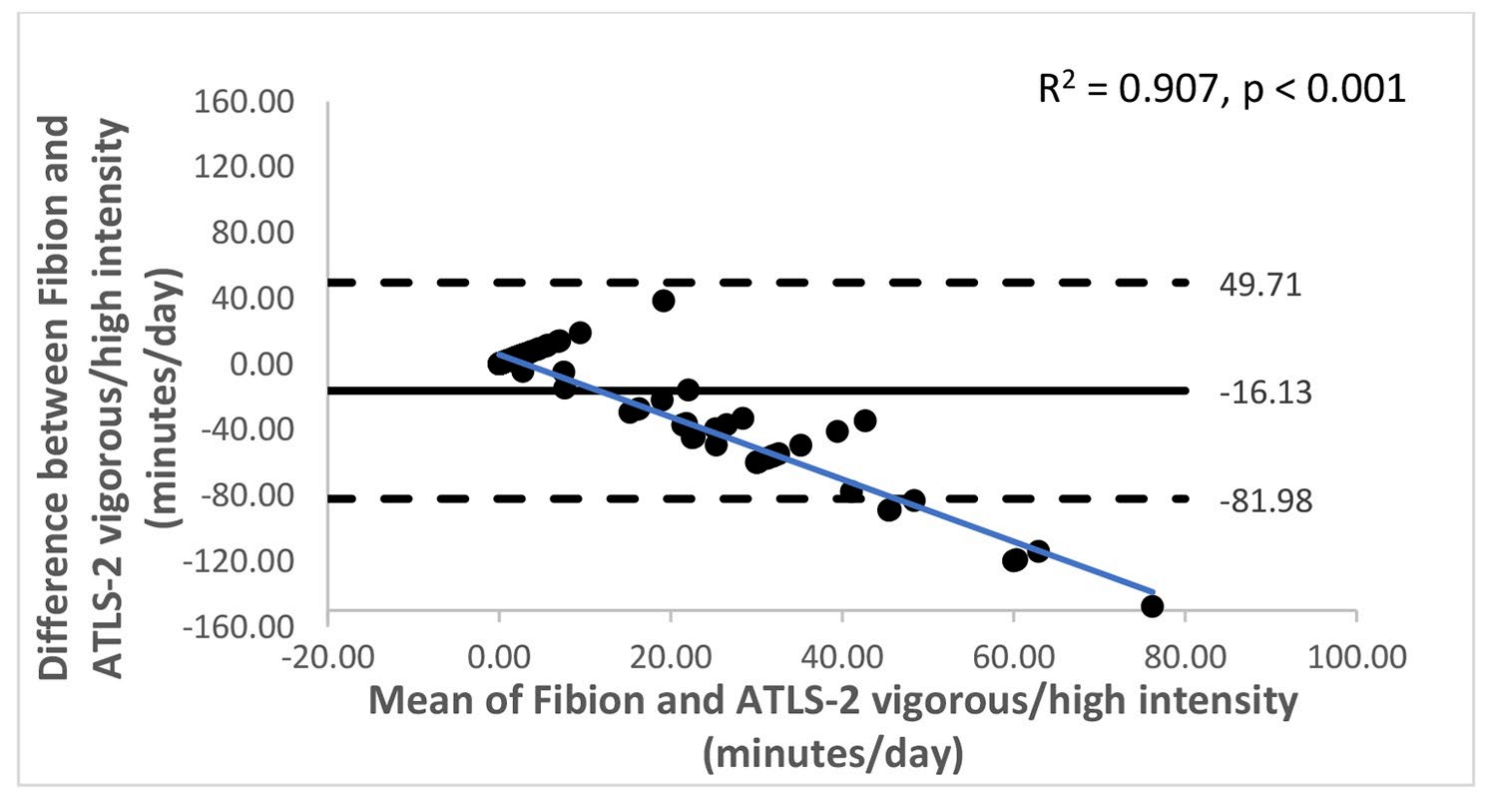
A Bland-Altman plot showing agreement between the Fibion accelerometer and ATLS-2 data for vigorous/high intensity activity time. The solid horizontal line represents bias (the mean of the differences between the two methods), while the horizontal dashed lines show upper and lower limits of agreement (bias ± (SD * 1.96)). The regression line is depicted in blue

**Fig. 5:**
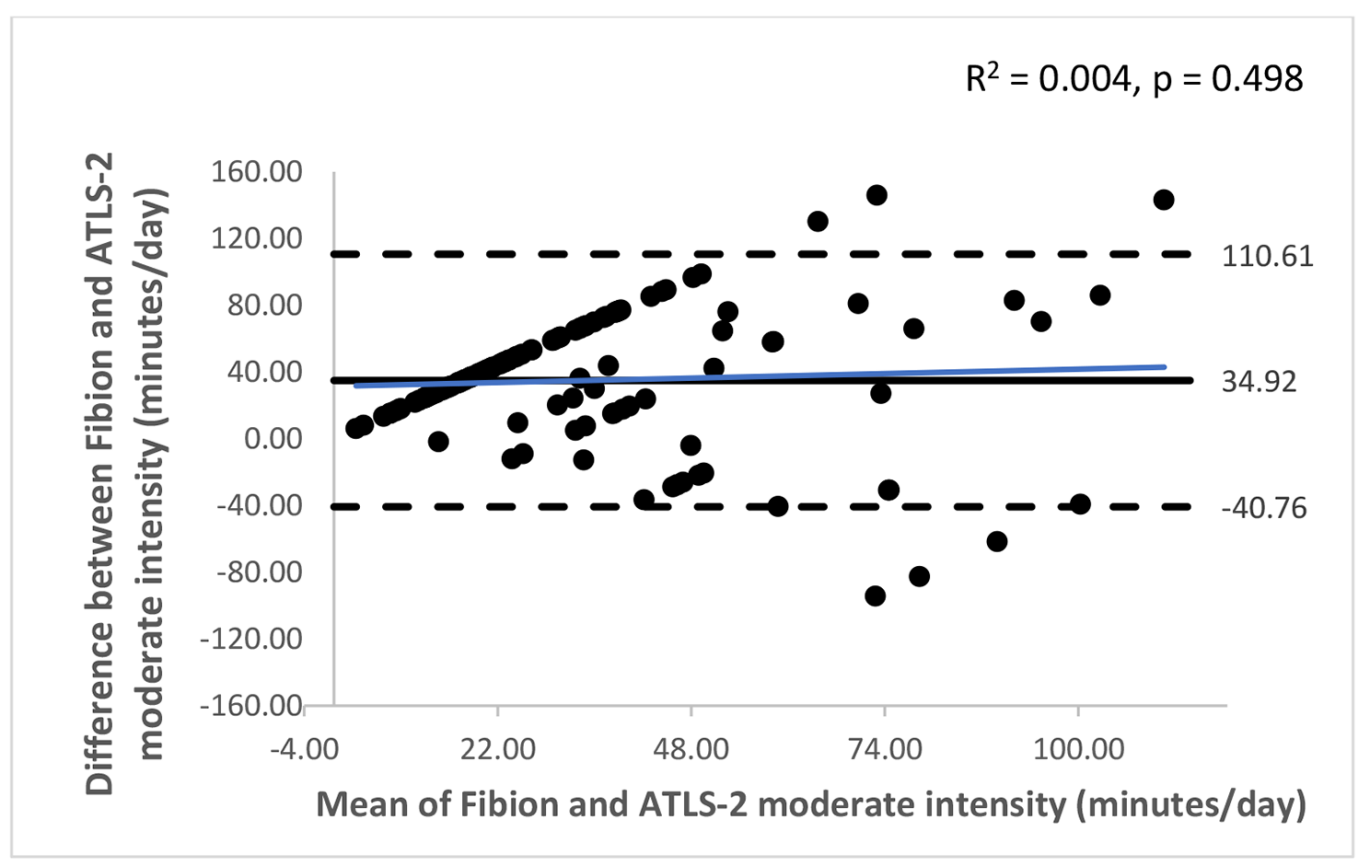
A Bland-Altman plot showing agreement between the Fibion accelerometer and ATLS-2 data for moderate intensity activity time. The solid horizontal line represents bias (the mean of the differences between the two methods), while the horizontal dashed lines show upper and lower limits of agreement (bias ± (SD * 1.96)). The regression line is depicted in blue

**Fig. 6:**
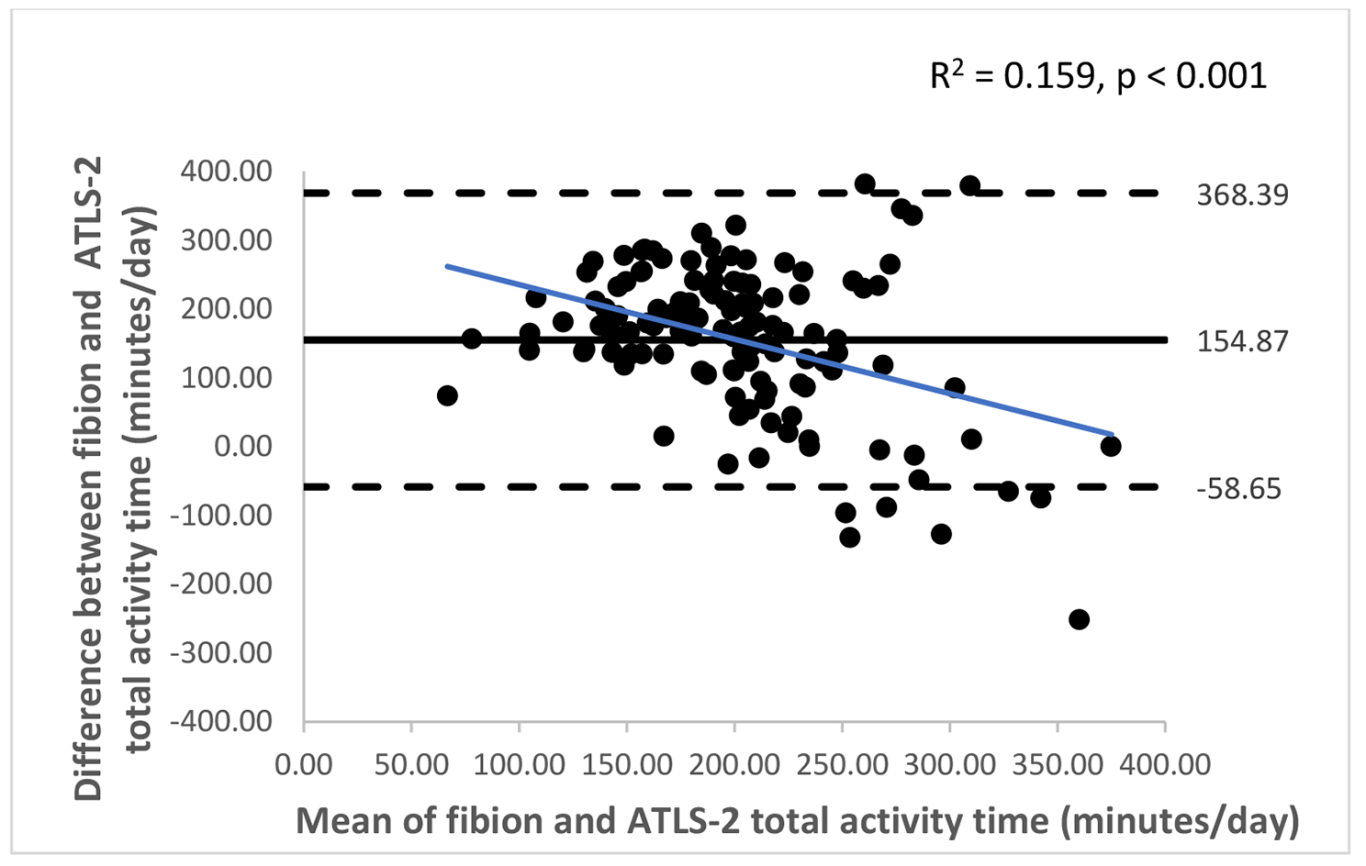
A Bland-Altman plot showing agreement between the Fibion accelerometer and ATLS-2 data for total activity time. The solid horizontal line represents bias (the mean of the differences between the two methods), while the horizontal dashed lines show upper and lower limits of agreement (bias ± (SD * 1.96)). The regression line is depicted in blue

The Bland-Altman plots with 95% limits of agreement are shown in Figs. 1, 2, 3, 4, 5 and 6. Except for walking and moderate intensity activity time (regression model p value > 0.05; Figs. 2 and 5), a proportional/systematic bias was evident in other plots (regression models p values < 0.05) with a tendency for a decrease in difference scores when there is an increase in mean scores between the methods as revealed by the regression lines.

## Discussion

This study investigated the concurrent validity of self-reported ATLS-2 and Fibion accelerometer-measured time during various activities performed during the day such as sitting, walking, cycling, high intensity and moderate intensity activities. Overall, there were negligible or weak correlations between most variables measured by the two methods. The findings from the study were consistent with our hypothesis. Subjective self-reported measures of the ATLS-2 were not as accurate as objectively measured sedentary and physical activity time parameters of the Fibion accelerometer, the reference standard used in our study. As the ATLS-2 is a self-reported measure, people often may not recall exactly how long they may be participating in certain activities, which may lead to an underestimation/overestimation of values. For example, it may be difficult to estimate how many hours they are sitting down during the day. Moreover, adolescents and young adults might underestimate or exaggerate their physical activity status when asked. These reasons can account for discrepancies in the results between the ATLS-2 questionnaire and the Fibion device measurements.

Specifically, the results showed a negligible correlation between the two methods for sitting/sedentary behavior (r = -0.04) and walking time (r = 0.05). The ATLS-2 seemed to underestimate sedentary time values and other variables (Table 3). Moreover, the participants in our study were teenagers and young adults (aged 14–25 years) and they were studying in schools/universities or were newly employed in jobs where they were required to sit for long periods of time. These participants may not be able to accurately estimate how long they have been sitting or doing other activities during the day depending on the variations in their day-to-day tasks and recall bias. The ATLS-2 questionnaire included questions on time spent for watching television, using internet and sleeping hours during weekdays and weekend days to record the sedentary behavior. This could not possibly account for all the sedentary activities people participate in as there are many other activities that can be considered sedentary such as sitting with family or reading. This would further contribute to underestimation of sitting/sedentary time.

A systematic review by Lee et al. (2011) found comparable results for self-reported walking time with the International Physical Activity Questionnaire short form (IPAQ-SF) [46]. When the IPAQ-SF was compared to different actometers, accelerometers and pedometers readings, they discovered that walking time was 28% underestimated at the level of smallest discrepancy. The problem this study highlights is that (brisk) walking frequently matches moderate intensity activity (walking: 3.3 metabolic equivalents [METs] and moderate intensity: 3-5.9 METs). Hence, the accelerometers might include brisk walking under moderate intensity activities. When compared to the accelerometers, the moderate intensity time calculated from the IPAQ-SF revealed weak relationships. Similar discrepancies are plausible while comparing ATLS-2 and Fibion accelerometer measurements.

A study by Wang et al. (2013) found that moderate physical activity levels of Chinese youth were overestimated by 106% with the IPAQ-SF when compared to ActiGraph measurements [28]. Therefore, the results for self-reported moderate intensity are different when compared to accelerometers, thereby posing doubt on the validity of self-reported sedentary and physical activity measures with the IPAQ-SF and ATLS-2.

A negligible correlation (r = -0.001) was found between high intensity activity time between both methods in our study. People usually participate in high-intensity sports for a short period of time but still correlations between self-reported and accelerometer-measured time were negligible. In a study by Al Hazza et al. in 2011, similar correlation estimates were found between the pedometer and ATLS-1 questionnaire [29]. The strength of correlation between the ATLS-1 and pedometer was weak (r = 0.338) for high-intensity ambulatory activities such as running and jogging and negligible for non-ambulatory activities like bicycling (r = 0.135), house-hold chores (r = 0.137), and weight training (r = 0.042). The results may not be directly comparable with our study as they used a pedometer and ATLS-1 whereas we used the Fibion accelerometer and ATLS-2. Nonetheless, in agreement with our findings, the strength of correlation between self-reported ATLS-1 and pedometer values was found to be weak or negligible (Table 1).

### Strengths, methodological considerations, and limitations

This is the first study to assess concurrent validity of the ATLS-2 physical activity questionnaire (Arabic and English versions) and a valid Fibion accelerometer in the United Arab Emirates. For this purpose, we included a large cohort of 131 healthy adolescents and young adults with valid Fibion accelerometer data of ≥ 600 min/days for at least 4 out of 7 days including three weekdays and one weekend day. Participants were asked to recall duration of specific activities of daily living during a typical (usual) week, which could have resulted in recall bias confounding the findings. Recall bias is not the only limitation with such questions but the ability of participants to accurately denote a duration to their activities could be quite difficult. The ATLS-2 questionnaire does not include a specific question inquiring about the number of hours a teen/young adult remain sedentary/sitting during a day. Most of the participants did not self-report to engage in many of the physical activities mentioned in the ATLS-2, such as biking, dancing, and sports of various intensities. Thus, physical activity of adolescents and young adults should be directly monitored with reliable and valid accelerometers or other similar devices, whenever possible, to get better interpretation of their sedentary behavior and physical activity [47]. This study mainly included a combination of school and university students. Future studies could exclusively validate the ATLS-2 physical activity questionnaire with accelerometers only in school children to see whether it yields more concordant findings between the two methods. However, further revision of the questionnaire of the ATLS-2 physical activity questionnaire is recommended.

As people in the United Arab Emirates live in a very dry and hot climate, most children/adolescents and young adults might not choose to do outdoor activities during daytime. Most extra-curricular activities are indeed not included in regular physical education classes for children. Thus, strikingly most participants did not score any value for most activities in the ATLS-2. However, the Fibion accelerometer device captured the duration of activities with different intensities irrespective of the type of task (during study, work or leisure). Participants were asked to remove the Fibion during any water-based activities which precluded comparison to swimming time collected in the ATLS-2.

We used the Fibion data fixer tool to exclude standard nighttime (11 pm to 7 am) from all participants to prevent conflation between nighttime data with sedentary and upright time data [37]. A few minutes of cycling data were recorded by the Fibion device for some participants even though those participants did not report cycling with the ATLS-2. Inaccurate classification of an activity may be caused by a device malfunction, such as a failure to detect signals or start-up issues, which may have an impact on the recording and processing of data. The self-reported scores with the ATLS-2 questionnaire underestimated the duration of sitting/sedentary time and physical activities of different type/intensity, despite the tasks’ duration reported for a 24-hour day, compared to corresponding Fibion data normalized to 16-hour day. Therefore, comparing these two outcomes (self-reported time per 24-hour day vs. Fibion-accelerometer data normalized to 16-hour day) is challenging yet relevant as Fibion data without normalization to 16-hour day would be confounded by device wear time of participants. However, these differences (self-reported ATLS-2 vs. normalized Fibion data) were not expected to confound the correlation values reported in the study.

### Future recommendations

The ATLS-2 questionnaire could add a distinct question about sitting time per day (in hours), to more specifically investigate this component. The number of physical activities included in the questionnaire is extensive and future studies must note that all tasks may not be applicable for all adolescents and young adults. Further validation of dietary component of the questionnaire is warranted. A waterproof wrap or sealed envelope enclosing the Fibion device could be used to allow participants to participate in water-based activities without having to remove device, therefore giving a chance to capture relevant data.

## Conclusion

This study showed an overall poor correlation and low agreement between self-reported ATLS-2 and Fibion accelerometer-measured sedentary and physical activity time. Negligible to weak correlations were noted for sitting, walking, cycling, high and moderate intensity activity and total activity time between the two methods. The ATLS-2 physical activity questionnaire may be used while collecting self-reported data on a large Arab (teen/young adult) population and in those settings where accelerometers are unavailable; however, the chances for underestimation of sedentary and physical activity time with such self-reported physical activity questionnaires must be noted. We recommend including accelerometers or similar devices whenever possible to provide objective physical activity estimates.

## Data Availability

Data are available from AA upon reasonable request.

## List of Abbreviations

ATLS: Arab Teen Lifestyle Study
ATLS-2: Revised version of the Arab Teen Lifestyle Study questionnaire
IPAQ-SF: International Physical Activity Questionnaire Short Form
METs: Metabolic equivalents
UAE: United Arab Emirates

## References

[1] O’Donovan G, Lee I-M, Hamer M, Stamatakis E. Association of ‘Weekend Warrior’ and other Leisure Time physical activity patterns with risks for All-Cause, Cardiovascular Disease, and Cancer Mortality. JAMA Intern Med. Mar. 2017;177(3):335. 10.1001/jamainternmed.2016.8014. 10.1001/jamainternmed.2016.8014 28097313

[2] Ibrahim B, Osman A (2019). Effect of physical activity status and dietary habits on pulmonary functions. J Mol Pathophysiology.

[3] Osman AA, Abumanga ZM. “The Relationship Between Physical Activity Status and Dietary Habits with the Risk of Cardiovascular Diseases,” *e-Journal of Cardiovascular Medicine*, vol. 7, no. 2, pp. 72–78, Jun. 2019, doi: 10.32596/ejcm.galenos.2019.00008.

[4] Alzamil H, Alhakbany M, Alfadda N, Almusallam S, Al-Hazzaa H. A profile of physical activity, sedentary behaviors, sleep, and dietary habits of saudi college female students. J Family Community Med. 2019;26(1). 10.4103/jfcm.JFCM_58_18. 10.4103/jfcm.JFCM_58_18 PMC6335843 30697098

[5] Al-Hazzaa HM. “Physical inactivity in Saudi Arabia revisited: a systematic review of inactivity prevalence and perceived barriers to active living.,” Int J Health Sci (Qassim), vol. 12, no. 6, pp. 50–64. PMC6257875 30534044

[6] Zaabi M, Shah SM, Sheek-Hussein M, Abdulle A, Junaibi A, Loney T. Results from the United Arab Emirates’ 2016 Report Card on Physical Activity for Children and Youth. J Phys Act Health. Nov. 2016;13:S299–S306. 10.1123/jpah.2016-0312. no. s2. 10.1123/jpah.2016-0312 27848750

[7] Paulo MS et al. “Results From the United Arab Emirates’ 2018 Report Card on Physical Activity for Children and Youth,” *J Phys Act Health*, vol. 15, no. s2, pp. S419–S421, Jan. 2018, doi: 10.1123/jpah.2018-0543. 10.1123/jpah.2018-0543 30475123

[8] Brickwood K-J, Watson G, O’Brien J, Williams AD. Consumer-based wearable activity trackers increase physical activity participation: systematic review and Meta-analysis. JMIR Mhealth Uhealth. Apr. 2019;7(4):e11819. 10.2196/11819. 10.2196/11819 PMC6484266 30977740

[9] Farnell G, Barkley J. The effect of a wearable physical activity monitor (Fitbit one) on physical activity behaviour in women: a pilot study. J Hum Sport Exerc. 2017;12(4). 10.14198/jhse.2017.124.09.

[10] Cole TJ, Lobstein T. Extended international (IOTF) body mass index cut-offs for thinness, overweight and obesity. Pediatr Obes. Aug. 2012;7(4):284–94. 10.1111/j.2047-6310.2012.00064.x. 10.1111/j.2047-6310.2012.00064.x 22715120

[11] Cole TJ, Flegal KM, Nicholls D, Jackson AA. Body mass index cut offs to define thinness in children and adolescents: international survey. BMJ. Jul. 2007;335(7612):194. 10.1136/bmj.39238.399444.55. 10.1136/bmj.39238.399444.55 PMC1934447 17591624

[12] Kallio J et al. “Changes in physical activity and sedentary time during adolescence: Gender differences during weekdays and weekend days,” *Scand J Med Sci Sports*, vol. 30, no. 7, pp. 1265–1275, Jul. 2020, doi: 10.1111/sms.13668. 10.1111/sms.13668 PMC7318293 32248577

[13] Nyberg G, Kjellenberg K, Fröberg A, Lindroos AK. “A national survey showed low levels of physical activity in a representative sample of Swedish adolescents,” *Acta Paediatr*, vol. 109, no. 11, pp. 2342–2353, Nov. 2020, doi: 10.1111/apa.15251. 10.1111/apa.15251 32266736

[14] Farooq A, et al. Longitudinal changes in moderate-to‐vigorous‐intensity physical activity in children and adolescents: a systematic review and meta‐analysis. Obes Rev. Jan. 2020;21(1). 10.1111/obr.12953. 10.1111/obr.12953 PMC6916562 31646739

[15] Ammar A, et al. Effects of COVID-19 Home Confinement on eating Behaviour and physical activity: results of the ECLB-COVID19 International Online Survey. Nutrients. May 2020;12(6):1583. 10.3390/nu12061583. 10.3390/nu12061583 PMC7352706 32481594

[16] Hourani H, Alkhatib B, Abdullah M. Impact of COVID-19 Lockdown on Body Weight, Eating Habits, and physical activity of jordanian children and adolescents. Disaster Med Public Health Prep. Feb. 2021;1–9. 10.1017/dmp.2021.48. 10.1017/dmp.2021.48 PMC8129676 33588981

[17] Abed Alah M, Abdeen S, Kehyayan V, Bougmiza I. “Impact of staying at home measures during COVID-19 pandemic on the lifestyle of Qatar’s population: Perceived changes in diet, physical activity, and body weight,” *Prev Med Rep*, vol. 24, p. 101545, Dec. 2021, doi: 10.1016/j.pmedr.2021.101545. 10.1016/j.pmedr.2021.101545 PMC8413097 34493967

[18] Alghadir A, Iqbal Z, Gabr SA. The Relationships of watching Television, Computer Use, Physical Activity, and Food Preferences to Body Mass Index: gender and nativity differences among adolescents in Saudi Arabia. Int J Environ Res Public Health. Sep. 2021;18(18):9915. 10.3390/ijerph18189915. 10.3390/ijerph18189915 PMC8469276 34574844

[19] Gallo LA, Gallo TF, Young SL, Moritz KM, Akison LK. The impact of isolation measures due to COVID-19 on Energy Intake and physical activity levels in australian University students. Nutrients. Jun. 2020;12(6):1865. 10.3390/nu12061865. 10.3390/nu12061865 PMC7353248 32585830

[20] Sharara E, Akik C, Ghattas H, Makhlouf Obermeyer C. Physical inactivity, gender and culture in arab countries: a systematic assessment of the literature. BMC Public Health. Dec. 2018;18(1):639. 10.1186/s12889-018-5472-z. 10.1186/s12889-018-5472-z PMC5960209 29776343

[21] Al-Hazzaa HM, Al-Sobayel HI, Musaiger AO. “Convergent Validity of the Arab Teens Lifestyle Study (ATLS) Physical Activity Questionnaire,” *Int J Environ Res Public Health*, vol. 8, no. 9, pp. 3810–3820, Sep. 2011, doi: 10.3390/ijerph8093810. 10.3390/ijerph8093810 PMC3194119 22016718

[22] Al-Hazzaa HM, Alothman SA, Alghannam AF, Almasud AA. “Anthropometric Measurements, Sociodemographics, and Lifestyle Behaviors among Saudi Adolescents Living in Riyadh Relative to Sex and Activity Energy Expenditure: Findings from the Arab Teens Lifestyle Study 2 (ATLS-2),” *Nutrients*, vol. 14, no. 1, p. 110, Dec. 2021, doi: 10.3390/nu14010110. 10.3390/nu14010110 PMC8746972 35010983

[23] Alhakbany MA, Alzamil HA, Alabdullatif WA, Aldekhyyel SN, Alsuhaibani MN, Al-Hazzaa HM (2018). Lifestyle Habits in relation to overweight and obesity among saudi women attending Health Science Colleges. J Epidemiol Glob Health.

[24] Al-Hazzaa H, Musaiger A. Arab teens Lifestyle Study (ATLS): objectives, design, methodology and implications. Diabetes Metab Syndr Obes. p. Dec. 2011;417. 10.2147/DMSO.S26676. 10.2147/DMSO.S26676 PMC3257970 22253540

[25] Khalaf A, Ekblom Ö, Kowalski J, Berggren V, Westergren A, Al-Hazzaa H. “Female University Students’ Physical Activity Levels and Associated Factors—A Cross-Sectional Study in Southwestern Saudi Arabia,” *Int J Environ Res Public Health*, vol. 10, no. 8, pp. 3502–3517, Aug. 2013, doi: 10.3390/ijerph10083502. 10.3390/ijerph10083502 PMC3774451 23939387

[26] Albawardi NM, AlTamimi AA, AlMarzooqi MA, Alrasheed L, Al-Hazzaa HM. “Associations of Body Dissatisfaction With Lifestyle Behaviors and Socio-Demographic Factors Among Saudi Females Attending Fitness Centers,” *Front Psychol*, vol. 12, Feb. 2021, doi: 10.3389/fpsyg.2021.611472. 10.3389/fpsyg.2021.611472 PMC7904690 33643138

[27] Skender S, et al. Accelerometry and physical activity questionnaires - a systematic review. BMC Public Health. Dec. 2016;16(1):515. 10.1186/s12889-016-3172-0. 10.1186/s12889-016-3172-0 PMC4910242 27306667

[28] Wang C, Chen P, Zhuang J. Validity and reliability of International Physical Activity Questionnaire–Short Form in Chinese Youth. Res Q Exerc Sport. Dec. 2013;84:S80–6. 10.1080/02701367.2013.850991. no. sup2. 10.1080/02701367.2013.850991 24527570

[29] Pesola AJ, Laukkanen A, Heikkinen R, Sipilä S, Sääkslahti A, Finni T. Accelerometer-assessed sedentary work, leisure time and cardio-metabolic biomarkers during one year: effectiveness of a cluster randomized controlled trial in parents with a sedentary occupation and young children. PLoS ONE. Aug. 2017;12(8):e0183299. 10.1371/journal.pone.0183299. 10.1371/journal.pone.0183299 PMC5570316 28837598

[30] Yang Y, Schumann M, Le S, Cheng S. Reliability and validity of a new accelerometer-based device for detecting physical activities and energy expenditure. PeerJ. Oct. 2018;6:e5775. 10.7717/peerj.5775. 10.7717/peerj.5775 PMC6186411 30324032

[31] DAVIES G, REILLY JJ, MCGOWAN AJ, DALL PM, GRANAT MH, PATON JY. Validity, practical utility, and reliability of the activPAL™ in Preschool Children. Med Sci Sports Exerc. Apr. 2012;44(4):761–8. 10.1249/MSS.0b013e31823b1dc7. 10.1249/MSS.0b013e31823b1dc7 21983077

[32] Basterfield L, et al. Physical activity, diet and BMI in children aged 6–8 years: a cross-sectional analysis. BMJ Open. Jun. 2014;4(6):e005001–1. 10.1136/bmjopen-2014-005001. 10.1136/bmjopen-2014-005001 PMC4054632 24902732

[33] Kridli SA-O, Liu Y-W, Bates A, Jilian M, Hayes D, Jaber LA. Do teen mentors improve the effectiveness of a culturally-adapted lifestyle intervention in arab american youth? A randomized, controlled study. J Nurs Educ Pract. Feb. 2017;7(7):119. 10.5430/jnep.v7n7p119.

[34] Dalibalta S et al. “Objectively quantified physical activity and sedentary behaviour in a young UAE population,” *BMJ Open Sport Exerc Med*, vol. 7, no. 1, p. e000957, Jan. 2021, doi: 10.1136/bmjsem-2020-000957. 10.1136/bmjsem-2020-000957 PMC7797257 33489309

[35] MCCLAIN JJ, SISSON SB, TUDOR-LOCKE C. “Actigraph Accelerometer Interinstrument Reliability during Free-Living in Adults,” *Med Sci Sports Exerc*, vol. 39, no. 9, pp. 1509–1514, Sep. 2007, doi: 10.1249/mss.0b013e3180dc9954. 10.1249/mss.0b013e3180dc9954 17805082

[36] Arumugam A et al. “Does Google Fit provide valid energy expenditure measurements of functional tasks compared to those of Fibion accelerometer in healthy individuals? A cross-sectional study,” *Diabetes & Metabolic Syndrome: Clinical Research & Reviews*, vol. 15, no. 6, p. 102301, Nov. 2021, doi: 10.1016/j.dsx.2021.102301. 10.1016/j.dsx.2021.102301 34592530

[37] Alsamman RA, Pesola AJ, Shousha TM, Hagrass MS, Arumugam A. Effect of night-time data on sedentary and upright time and energy expenditure measured with the Fibion accelerometer in emirati women. Diabetes & Metabolic Syndrome: Clinical Research & Reviews. Feb. 2022;16(2):102415. 10.1016/j.dsx.2022.102415. 10.1016/j.dsx.2022.102415 35104752

[38] Alkalih HY, Pesola AJ, Arumugam A. A new accelerometer (Fibion) device provides valid sedentary and upright time measurements compared to the ActivPAL4 in healthy individuals. Heliyon. 2022 Oct 1;8(10):e11103. 10.1016/j.heliyon.2022.e11103 PMC9586911 36281387

[39] Terwee CB et al. “COSMIN methodology for assessing the content validity of PROMs user manual version 1.0.” [Online]. Available: www.cosmin.nl.

[40] Anthoine E, Moret L, Regnault A, Sébille V, Hardouin J-B. Sample size used to validate a scale: a review of publications on newly-developed patient reported outcomes measures. Health Qual Life Outcomes. Dec. 2014;12(1). 10.1186/s12955-014-0176-2. 10.1186/s12955-014-0176-2 PMC4275948 25492701

[41] Al-Hazzaa H. “Arab Teens Lifestyle (ATLS) Questionnaire (Revised 2018),” https://lh-hsrc.pnu.edu.sa/wp-content/uploads/2018/11/ATLS-Questionnaire-E-Revised-2018-1-1.pdf

[42] Nirhamo E. “The Impact of the Fibion Change Program on Sitting and Activity Habits: A Three-Month Occupational Well-Being Intervention.,” *Faculty of Sport and Health Sciences, University of Jyväskylä, Master’s thesis in Sports and Exercise Medicine*, p. 55, 2021.

[43] HEALY GN et al. “A Cluster Randomized Controlled Trial to Reduce Office Workers’ Sitting Time,” *Med Sci Sports Exerc*, vol. 48, no. 9, pp. 1787–1797, Sep. 2016, doi: 10.1249/MSS.0000000000000972. 10.1249/MSS.0000000000000972 27526175

[44] Schober P, Boer C, Schwarte LA. Correlation coefficients. Anesth Analg. May 2018;126(5):1763–8. 10.1213/ANE.0000000000002864. 10.1213/ANE.0000000000002864 29481436

[45] Tinsley GM. “Proportional bias between dual-energy x-ray absorptiometry and bioelectrical impedance analysis varies based on sex in active adults consuming high- and low-carbohydrate diets,” *Nutrition Research*, vol. 42, pp. 85–100, Jun. 2017, doi: 10.1016/j.nutres.2017.05.003. 10.1016/j.nutres.2017.05.003 28633874

[46] Lee PH, Macfarlane DJ, Lam T, Stewart SM. “Validity of the international physical activity questionnaire short form (IPAQ-SF): A systematic review,” *International Journal of Behavioral Nutrition and Physical Activity*, vol. 8, no. 1, p. 115, Dec. 2011, doi: 10.1186/1479-5868-8-115. 10.1186/1479-5868-8-115 PMC3214824 22018588

[47] Benítez-Porres J, López-Fernández I, Raya JF, Álvarez Carnero S, Alvero-Cruz JR, Carnero E. “Reliability and Validity of the PAQ-C Questionnaire to Assess Physical Activity in Children,” *Journal of School Health*, vol. 86, no. 9, pp. 677–685, Sep. 2016, doi: 10.1111/josh.12418. 10.1111/josh.12418 27492937

